# Assessment of Harbour Porpoise Bycatch along the Portuguese and Galician Coast: Insights from Strandings over Two Decades

**DOI:** 10.3390/ani13162632

**Published:** 2023-08-15

**Authors:** Andreia Torres-Pereira, Hélder Araújo, Silvia Silva Monteiro, Marisa Ferreira, Jorge Bastos-Santos, Sara Sá, Lídia Nicolau, Ana Marçalo, Carina Marques, Ana Sofia Tavares, Myriam De Bonis, Pablo Covelo, José Martínez-Cedeira, Alfredo López, Marina Sequeira, José Vingada, Catarina Eira

**Affiliations:** 1 Department of Biology & ECOMARE/CPRAM, Universidade de Aveiro, 3810-193 Aveiro, Portugal; helder.araujo@ua.pt (H.A.); s.monteiro@ua.pt (S.S.M.); saramsa@ua.pt (S.S.); sofia19@ua.pt (A.S.T.); myriam.bonis@ua.pt (M.D.B.); a.lopez@ua.pt (A.L.); catarina.eira@ua.pt (C.E.); 2 Centre for Environmental and Marine Studies (CESAM), Universidade de Aveiro, 3810-193 Aveiro, Portugal; 3 Portuguese Wildlife Society (SPVS), Estação de Campo de Quiaios, 3081-101 Figueira da Foz, Portugal; mctferreira@socpvs.org (M.F.); jorgeb.santos@gmail.com (J.B.-S.); lvrnicolau@gmail.com (L.N.); carina.marques@socpvs.org (C.M.); spvs@socpvs.org (J.V.); 4 Centre of Marine Sciences (CCMAR), Universidade do Algarve, Campus de Gambelas, FCT Ed. 7, 8005-139 Faro, Portugal; amarcalo@ualg.pt; 5 Coordinadora para o Estudio dos Mamíferos Mariños (CEMMA), Apdo., 15-36380 Gondomar, Spain; pablo_cov@yahoo.es (P.C.); jmcedeira@yahoo.es (J.M.-C.); 6 Instituto da Conservação da Natureza e Florestas (ICNF), Av. da República 16, 1050-191 Lisboa, Portugal; marina.sequeira@icnf.pt

**Keywords:** Iberian Peninsula, marine mammals, *Phocoena phocoena*, fisheries, potential biological removal, critically endangered

## Abstract

**Simple Summary:**

The Iberian harbour porpoise is currently threatened by accidental captures in fisheries (bycatch). Because monitoring cetacean bycatch is particularly challenging, marine mammal stranding networks may provide important information. Between 2000 and 2020, 756 porpoises washed ashore (stranded) on Portuguese and Galician coastlines. The post-mortem analyses of stranded porpoises revealed that the most representative cause of stranding (46.98% of the analysed porpoises) was fishery interaction and another 10.99% was identified as probable fishery interaction. Combining this information with porpoise annual abundance estimates in Portugal available for the period between 2011 and 2015, an estimated average of 207 porpoises died each year due to bycatch in Portuguese waters alone. This estimate greatly surpassed the maximum annual number of porpoise strandings due to human interactions (22 porpoises) that were predicted to occur without negatively affecting the population in Portuguese waters. To prevent porpoise bycatch in Portugal and Spain, fishing effort management is needed and new activities at sea must be carefully considered. Moreover, appropriate measures directed at the conservation of the Iberian harbour porpoise are crucial to ensure the restoration and survival of the population.

**Abstract:**

The Iberian harbour porpoise population is small and fisheries bycatch has been described as one of its most important threats. Data on harbour porpoise strandings collected by the Portuguese and Galician stranding networks between 2000 and 2020 are indicative of a recent mortality increase in the western Iberian coast (particularly in northern Portugal). Overall, in Portugal and Galicia, individuals stranded due to confirmed fishery interaction represented 46.98% of all analysed porpoises, and individuals stranded due to probable fishery interaction represented another 10.99% of all analysed porpoises. Considering the Portuguese annual abundance estimates available between 2011 and 2015, it was possible to calculate that an annual average of 207 individuals was removed from the population in Portuguese waters alone, which largely surpasses the potential biological removal (PBR) estimates (22 porpoises, CI: 12–43) for the same period. These results are conservative and bycatch values from strandings are likely underestimated. A structured action plan accounting for new activities at sea is needed to limit the Iberian porpoise population decline. Meanwhile, there is an urgent need for a fishing effort reorganization to directly decrease porpoise mortality.

## 1. Introduction

Recent genetic studies revealed that the harbour porpoise population in the upwelling areas of the Iberian Peninsula is small and isolated [1,2]. In fact, a new subspecies, *Phocoena phocoena meridionalis,* has already been proposed to include the so-called Iberian and Mauritanian harbour porpoise groups [1]. The small size of the Iberian population was confirmed in the European survey performed in 2016, which revealed a population estimate of 2898 porpoises (CV = 0.32) on the Atlantic coast of Portugal and Spain [3]. The harbour porpoise population is now critically endangered in Portugal [4] and endangered in Spain [5].

Fisheries bycatch has been described as the most important threat to small cetaceans [6,7,8], particularly in those countries where fishing is an important activity. Portugal and Spain have relatively large fishing fleets, including numerous small vessels that operate in coastal areas [9,10,11,12,13], leading to a higher encounter probability between fishing gear (particularly gill and trammel nets) and porpoises. There are several difficulties associated with applying direct and representative bycatch monitoring methods (e.g., attaining sufficient coverage with onboard observers in large fleets [14] or monitoring bycatch events in low abundance populations [15,16]). Therefore, marine mammal stranding networks have been increasingly viewed as a potential source of important information (such as cause of death and life history), while also allowing for relative population abundance estimates or spatio-temporal estimates of bycatch rates, (e.g., [17,18,19,20,21,22]).

However, estimating bycatch rates from strandings is also problematic. Cetaceans that die at sea do not necessarily strand ashore [23], and their probability of detection is influenced by several physical and biological factors, including proximity of the carcass to the shore, its buoyancy, decomposition rate, scavenging, and oceanographic and climatic processes, such as sea temperature, wind, and currents [20,24]. Additionally, it is currently accepted that in order to identify bycatch as the cause of death, specific post-mortem investigations are needed following an evidence-based medicine approach [25]. Also, more information can be retrieved from fresh to moderately decomposed carcasses than from decomposed individuals [26,27].

The objective of the present study was to assess the evolution of harbour porpoise strandings registered in Portugal and Galicia across two decades (2000–2020), particularly focussing on the temporal evolution of porpoises stranded due to fishery interaction. Finally, we also aimed to estimate an annual porpoise population removal rate based on fishery interactions related to mortality identified in porpoise strandings in Portugal.

## 2. Materials and Methods

### 2.1. Strandings in Western Iberia

#### 2.1.1. Data Collection

Data recorded between 2000 and 2020 by the National Strandings Network in Portugal (coordinated by the Instituto da Conservação da Natureza, ICNF) and the Galicia Strandings Network (coordinated by Xunta de Galicia and implemented by Coordinadora para o Estudio dos Mamíferos Mariños, CEMMA) were used in the present study.

Results concerning Portugal are divided between northern and southern sectors because the northern sector had a more constant and dedicated effort from the regional strandings network since the year 2000 (Sociedade Portuguesa de Vida Selvagem, SPVS), particularly between 2010 and 2020 (SPVS and University of Aveiro). The southern sector had a more dedicated effort in the Algarve region, between 2010 and 2017 (SPVS) and partly in 2020 (University of Algarve). In areas and periods with no dedicated effort, strandings were monitored by maritime police officers or park rangers. In Galicia, the strandings network’s effort was constant during the study period.

Both in Portugal and Galicia, detailed necropsies were performed by trained technicians and the cause of stranding was determined based on full external and internal examination [28]. Cause of stranding (including interaction with fisheries) was determined when: (1) strandings were attended by members of the stranding networks, and (2) individuals were found fresh or in a moderate decomposition stage (≤3 stages) [29,30], since an advanced decomposition state may mask evidence of the cause of stranding. Animals presenting unequivocal signs of interaction with fisheries (e.g., evident net marks) were considered as bycaught, whereas porpoises presenting some criteria indicative of interaction with fisheries (e.g., line marks + good nutritional status + whole or partially digested prey in stomach + bruising around appendages/neck + froth in the lungs) and no signs of other possible causes were considered as probably bycaught, based on the criteria described in the literature (see [31]). The category “others” includes interspecific attacks, asphyxia, dependent calves, and emaciation. Currently, it is accepted that forensic pathology methods are needed to ascertain the cause of death [25,32].

Neonates were identified by the presence of vibrissae, foetal folds, or umbilical cord, including cord scars [33]. Calves included individuals with a total body length (TBL) ≤ 125 cm (<one year old) [33]. Adult females were separated from juveniles based on previous maturity analysis. For those animals with no data on maturity, TBL was used as a proxy for age. Females were considered adults at TBL ≥ 168.9 cm [34] and males at TBL ≥ 151 cm [33]. Most strandings not attended by members of the stranding networks were not considered for the age class evaluation (neonates, calves, juveniles, and adults).

#### 2.1.2. Data Analysis

All strandings locations were plotted in QGIS 3.16.16-Hannover [35] using WGS 1984 and then projected using the metric Projection Coordinate System (ETRS89/Portugal TM06/EPSG:3763). Maps with a bathymetric gradient [36] showing a point density interpolation using the Kernel Density Estimation (KDE) tool with a 30 km radius were produced to visualize the areas with higher stranding densities (hotspots) for the overall period (2000–2020) and for each of the four considered periods (2000–2004, 2005–2009, 2010–2014, 2015–2020). The overall study period was divided into four subperiods to highlight possible changes over time.

Matrix heatmaps were built as an initial approach to visualize the monthly, seasonal and long-term temporal variations of the total number of harbour porpoise strandings per geographical area (Portugal and Galicia) using ggplot2 package version 3.4.2 [37] in R. Long-term temporal variation was assessed annually and for four different periods (2000–2004, 2005–2009, 2010–2014, 2015–2020). In order to visualise possible seasonal patterns in harbour porpoise strandings, four seasons were defined: winter (January–March), spring (April–June), summer (July–September) and autumn (October–December). A seasonal matrix heatmap by period was also built to visualize the bycatch information determined during the necropsies of stranded porpoises.

Generalized additive models (GAMs) were then used to evaluate the influence of explanatory variables on harbour porpoise strandings along the western Iberian coastline (Portugal and Galicia) between 2000 and 2020. All data series were explored for outliers, collinearity, and heterogeneity of variance and plotted for visualization of potential relationships between response and explanatory variables [38]. The number of strandings (response variable) was modelled as a function of month (short-term trends), year (long-term trends) and geographical area (Portuguese vs. Galician coasts). A negative binomial distribution was used to account for overdispersion. The geographical area was incorporated in the model as a categorical variable, while year and month were included as continuous variables. Month was included as a smoother since a non-linear effect was expected. The complexity of the smoother (knots) was automatically selected by the cross-validation function available within the mgcv package version 1.8-39 [39] in R. The final model was selected using a backwards model selection process. The model was identified based on the lowest Akaike information criterion (AIC) [40]. Model validation involved checking the assumptions of homogeneity, independence of residuals and lack of highly influential data points (‘hat’ values) [41]. Although interactions between temporal variables (especially month) and geographical areas were expected, the low sample size precluded the inclusion of interactions between variables in the model. Posterior GAM analyses were individually performed for strandings in Portugal and in Spain to detect potential temporal variation (month, year) in porpoise strandings per region. All analyses were performed in R v. 4.1.3 [42].

### 2.2. Bycatch Assessment and Potential Biological Removal: Insights from Portuguese Data

A deeper analysis was dedicated to porpoise strandings data in Portugal, particularly because porpoise annual population abundances were available from 2011 to 2015 [43] in Portugal but not in Galicia. The proportion of stranded bycaught animals (N_bycatch_) was estimated [23] and is most likely underestimated, since only fresh to moderately decomposed carcasses can be evaluated to determine the cause of death. Then, the carcass detection rate (CDR), the number of animals dying per year (estimated annual mortality (EAM)) and the percentage of animals removed from the population due to bycatch based on strandings data (annual population removal based on strandings data (APR_strandings_)) were also estimated based on the number of stranded porpoises evaluated for bycatch evidence (strandings), as described in Table S1. To obtain EAM, porpoise annual and overall abundance data (with respective confidence intervals) from 2011 to 2015 [43] and a specific mortality rate (Mr = 0.18) for the study area [33] were used. The estimated annual mortality due to bycatch (EAM_bycatch_), carcass detection rate (CDR, relevant for estimating EAM_bycatch_) and APR_strandings_ were also recalculated considering the total number of stranded porpoises instead of the number of stranded porpoises evaluated for bycatch evidence. Whereas using the total number of observed porpoise strandings to calculate EAM accounts for possible bycaught individuals which could not be evaluated for bycatch (decomposition stage > 3), a more accurate APR estimate can be obtained considering only the number of stranded porpoises which were evaluated for bycatch evidence, despite the lower number of individuals included in the analysis. All parameters were calculated annually and for the overall period (2011–2015). Estimated mortality rates and the average removal values are expressed as 95% confidence intervals.

It was also possible to estimate the potential biological removal (PBR) in Portuguese waters because population abundance estimates (*N*) and the respective coefficient of variation (CV*_N_*) were obtained from aerial surveys performed in Portuguese waters during the period 2011–2015 [43]. To estimate PBR (i.e., the number of animals that “may” be removed from a cetacean population, not including natural mortalities, without compromising the population at the biological level [44]), the following formula was used:

PBR = *N*_min_ 1/2 R_max_ *f*,

where *N*_min_ is the minimum population abundance estimate (lower 20th percentile of a log-normal distribution) estimated by:

*N*_min_ = *N*/exp [0.842 √ln(1 + CV_N_^2^)],

R_max_ is the maximum annual net recruitment rate; *f* is a recovery factor between 0.1 and 1.

The maximum net recruitment or population growth rate (R_max_) of 4% is the default value used for small cetaceans [44]. In order to provide conservative PBR estimates, the expected *f* value for depleted and threatened stocks and stocks of unknown status (0.5) was used instead of the *f* value for endangered or declining species (0.1) [45]. 

## 3. Results

### 3.1. Strandings in Western Iberia

Considering the overall dataset, between 2000 and 2020, 756 stranded harbour porpoises were reported on the western Iberian coast (comprising Portugal and Galicia) (Table 1). 

**Figure 1:**
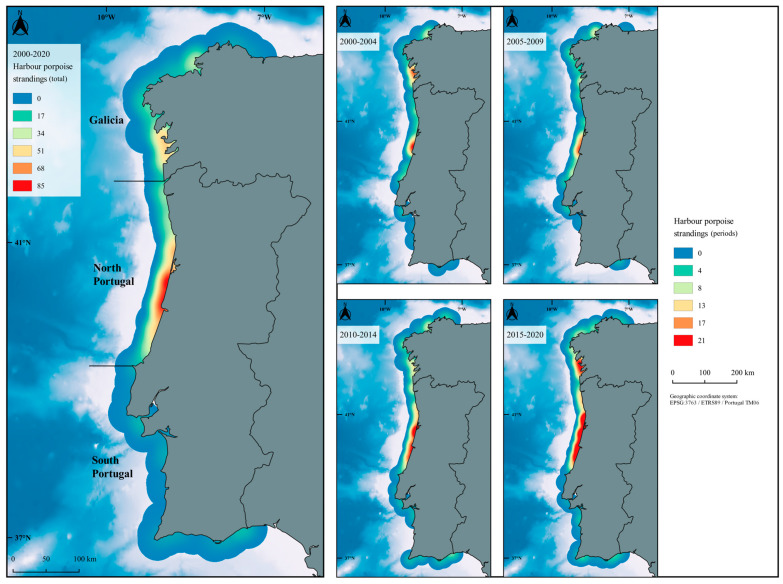
Harbour porpoise strandings in the western Iberian coast considering the overall study period (2000–2020) and four different periods (KDE, 30 km radius).

**Figure 2:**
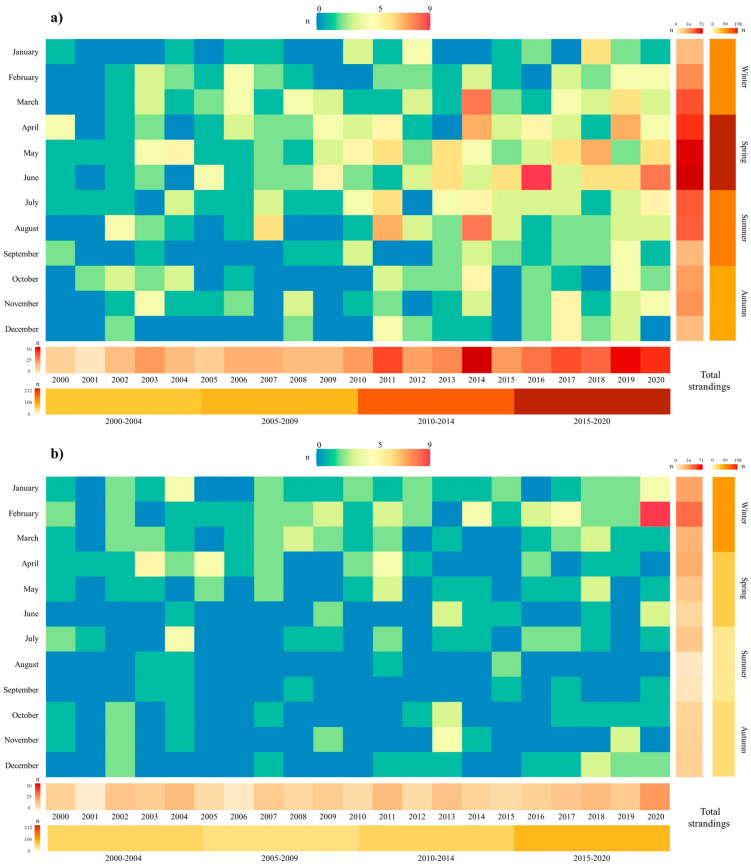
Matrix heatmaps for monthly porpoise strandings by year (column: years; row: months), total monthly and seasonal porpoise strandings (right side columns) and annual porpoise strandings and total porpoise strandings within the four considered study periods (bottom rows) (**a**) in Portugal; (**b**) and in Galicia.

**Figure 3:**
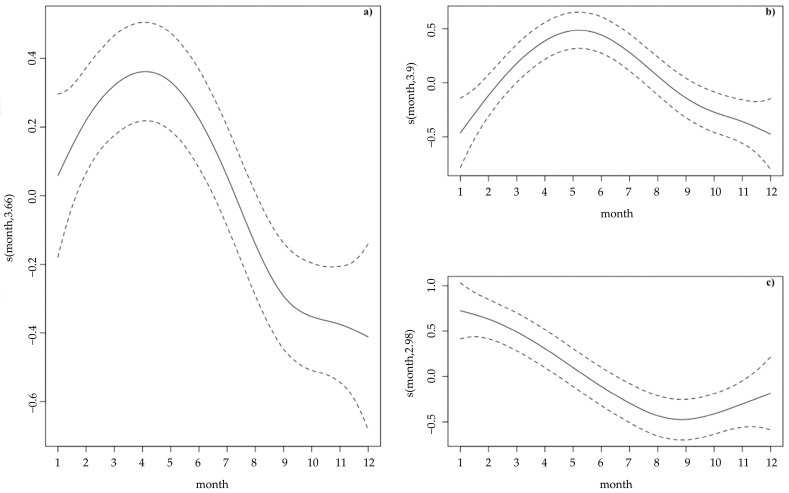
Smoothers for the seasonal effect (month) on the harbour porpoise strandings considering (**a**) the total strandings data; (**b**) strandings on the Portuguese coast and (**c**) strandings on the Galician coast. Dashed lines represent 95% confidence intervals.

GAM results emphasized a significant effect of month (*p* < 0.001), year (*p* < 0.001) and geographical area (*p* < 0.001) (r^2^ = 25.3%) on the number of porpoise strandings in the western Iberian coast. Based on the data analysed, in Portugal, the number of stranded porpoises (*n* = 524; coastal length = 835 km) was more than double the number of porpoises stranded in Galicia (*n* = 232; coastal length = 1190 km) during the study period. In Portugal, most porpoise strandings were registered in the northern sector (*n* = 453), while in Galicia the highest densities of strandings occurred in the southern Rias Baixas (*n* = 115) (Figure 1). The significant increase in porpoise strandings throughout the years in the western Iberian coast is particularly evident in the most recent period (2015–2020) (Figure 1 and Figure 2). With respect to the short-term pattern (monthly variations), a significant increase in strandings was detected during the first months of the year (peaking in March–May, Figure 3a).

GAM analyses performed separately for Portugal showed an effect of month (*p* < 0.001) and year (*p* < 0.001) (r^2^ = 28.8) on the number of porpoise strandings. On the Portuguese coast, the temporal trend in porpoise strandings was similar to the overall dataset with a significant increase over time (Figure 1) and also over the first half of the year, peaking in spring (particularly May and June, Figure 3a,b). More stranded porpoises were registered in March and August of 2014, and in June of 2016 and 2020 (Figure 2a).

GAM analyses performed separately for Galicia also showed a significant effect of month (*p* < 0.001) and year (*p* < 0.026) (r^2^ = 14.7%) on the number of porpoise strandings. Contrary to the monthly pattern observed in Portugal, the peak of porpoise strandings on the Galician coast occurred in winter (January and February), followed by a sharp decrease until September (Figure 3c). Regarding the effect of year, porpoise strandings increased on the Galician coast throughout the years, with a peak in 2020 (Figure 1 and Figure 2b).

Considering the total dataset, the observed proportions of females and males were 38.62% and 42.59%, respectively (Table 1). There was also a relatively high proportion of individuals that could not be sexed (18.78%).

Approximately 43% of the stranded harbour porpoises were juveniles, while 25% were adults (considering the 511 evaluated individuals, Table 1). Age class could not be determined for nearly half of the registered individuals. Seventeen foetuses were detected in females stranded in the northern region of Portugal, whereas no foetuses were detected in the southern sector and two were detected in Galicia (Table 1). Given the large number of individuals in an advanced decomposition state in Galicia (which were excluded from the present analysis), the number of foetuses might have been higher. 

Out of the 756 registered porpoise strandings, only 364 were considered for evaluation of the cause of stranding (Table 1). Both in Portugal and in Galicia, to provide more reliable evidence of fishery interaction in each stranding event, an important portion of stranded porpoises was excluded from the analysis (46.4% and 64.2%, respectively) mostly due to carcass decomposition state (in the northern sector of Portugal, only five porpoises were not examined due to “unavailable stranding team” or “inaccessible locations”). Overall, a total of 319 individuals were found in decomposition states 4 and 5 and another 73 were not evaluated (totalling 392 excluded individuals). It was not possible to determine the cause of stranding for a quarter of the analysed porpoises (25.55%) (Table 1).

**Figure 4:**
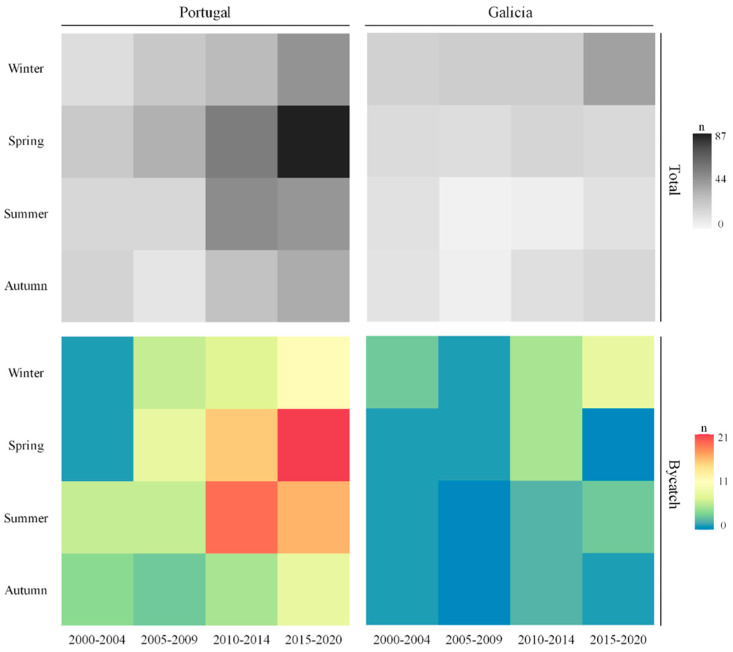
Matrix heatmaps for seasonal porpoise strandings by each of the four considered periods (column: periods; row: seasons) in Portugal and in Galicia. Total number of porpoise strandings (upper matrixes) and number of porpoises identified as stranded due to bycatch (lower matrixes).

Individuals stranded due to fishery interaction represented 46.98% of all analysed porpoises, and stranded individuals presenting probable evidence of fishery interaction represented another 10.99% of all analysed porpoises. Matrix heatmaps indicate that porpoises stranded due to fishery interaction increased throughout the studied periods, particularly in Portugal in spring and summer, and in Galicia in winter (Figure 4).

Emphasis is given to the northern region of Portugal, where 51.51% of the analysed porpoises were correlated with fishery interaction, and porpoises with evidence of possible fishery interaction represented another 12.12% out of the 264 analysed porpoises. In Galicia (Spain), 40.96% of the analysed porpoises presented evidence of fishery interaction, and porpoises showing probable evidence of fishery interaction represented another 7.23%, out of the 83 analysed porpoises.

### 3.2. Bycatch Assessment and Potential Biological Removal: Insights from Portuguese Data

When considering only the number of stranded porpoises which were evaluated for bycatch evidence and presented a decomposition stage ≤ 3, the annual average of individuals removed from the population between 2011 and 2015 (corresponding to the available annual population abundance estimates) amounts to 207 individuals, corresponding to an APR_strandings_ of 9.19% (CI = 5.25–16.10%) (Table 2). However, when using the total number of porpoises stranded in Portugal during the considered period, the theoretical minimum number of porpoises stranded due to bycatch was estimated to be, on average, 125 individuals per year (EAM_bycatch_), corresponding to an APR_strandings_ of 5.55% (CI = 3.17–9.72%) (Table S2).

For the period between 2011 and 2015, the overall PBR estimates indicate an “acceptable” average annual removal in Portuguese waters of 22 porpoises (CI: 12–43). The maximum annual PBR estimate of 29 porpoises (CI: 14–60) was obtained in 2013 (Table 3).

## 4. Discussion

The present study revealed that 46.98% of all analysed porpoises in Portugal and Galicia presented evidence of fishery interaction, and another 10.99% of all analysed porpoises presented probable evidence of fishery interaction. Also, we estimated that an annual average of 207 individuals were removed from the population in Portuguese waters alone between 2011 and 2015.

Considering the western Iberian Peninsula, over the last two decades, most harbour porpoise strandings were concentrated in the northern sector of the Portuguese coast and in the southern Rias Baixas in Galicia (Spain). The increasing incidence of porpoise strandings particularly during the last decade partly relates to the improved response of the national strandings network in Portugal, as already discussed in other regions [22,46,47]. Nevertheless, data clearly indicate an increase in porpoise strandings (including porpoises presenting evidence of fishery interaction) in Portugal and Galicia in the most recent period (2015–2020).

The number of strandings may be related to biotic (e.g., abundance, mortality rate or habitat use of the stranded species) or abiotic variables (e.g., mortality due to fisheries bycatch), but is also dependent on other factors such as stranding probability and detection rate, which in turn are influenced by environmental variables (e.g., weather and oceanographic features, coast physical characteristics) [20,24]. A future analysis involving all the above-mentioned variables would be important to clarify the statistical meaning of the patterns observed on stranding records, namely the higher number of porpoise strandings in Portuguese areas that occur in spring (May and June), whereas, in Galicia, the peak occurs, so far, in winter (February 2020). 

As an example of the potential effect of different variables on porpoise strandings, the highest number of porpoises stranded in Portugal in 2014 (particularly in March and April and then in August) may be related to the higher porpoise abundances estimated in October 2013 [43]. However, the significance of this record needs further evaluation as more detailed abundance estimates and changes in distribution (e.g., seasonal and annual) have never been assessed. The higher observed number of total strandings in Portugal during spring and summer may be linked with the species’ use of space in relation to their reproduction cycle and prey distribution. An increased number of observers in the summer months may also contribute to stranding reports in this season. However, it also seems to be related to fishing using gill/trammel nets, beach seines and possibly illegal, unreported and unregulated (IUU) fisheries. Based on fisheries observers, voluntary logbooks and remote electronic monitoring data collected during project LIFE+ MarPro [48], fleets using gill/trammel nets and beach seines showed considerable bycatch rates in Portugal. The use of gill and trammel nets is very intensive both in Portugal and Galicia. Recent data indicated 3783 artisanal fishing vessels registered in Galicia [49]. In 2020, there were 1820 boats in Galicia licensed to use bottom gillnets, including 1502 boats (mostly <10 m, 84%) registered in harbours located within a critical area proposed for porpoises [50]. Also, project VIRADA (monitoring small-scale fisheries using bottom set nets) estimated that 48.4% of 184 monitored fishing events used illegal practices according to Decree 115/2011 of the Galician Autonomous Community [51]. In Portugal, around 90% of the fleet comprises small polyvalent (multi-gear) fishing vessels using gill and trammel nets [52]. In 2020, a total of 5054 gill and trammel net licences were issued on the continental Portuguese coast, mostly (88%) allocated to boats smaller than 10 m [51] that operate near the coast. On the other hand, although beach seines represent a very small number of boats (annually, <40 licences), they concentrate in a relatively small area (a 115 km long coastline) which overlaps the region where most strandings were detected over the years (Figure 1). Beach seines are carried out to sea by a small vessel and hauled back from the beach [53] by metal net haulers coupled to tractors. The typical beach seine activity during spring and summer months adds to the already-elevated fishing pressure, thus probably contributing to the higher number of strandings in those seasons. 

A concerning amount of porpoises stranded with evidence of fishery interaction in the western Iberia area (46.98%) emphasises the need for bycatch mitigation measures to decrease porpoise mortality. Note that another 10.99% were conservatively identified as stranded due to probable fishery interaction and, furthermore, over half of the stranded porpoises were not considered in the cause of stranding evaluation to avoid inaccurate identification of fishery interaction. Therefore, the global estimate (~47%) is a minimum value for porpoises stranded with evidence of fishery interaction in Portugal and Galicia. It is also noteworthy that when only considering the 264 porpoises analysed for their cause of stranding in the northern region of Portugal (where the largest number of strandings were registered), the proportion of porpoises with evidence of fishery interaction rises to 51.51%. If individuals demonstrating probable fishery interaction were considered, the proportion of porpoises interacting with fisheries in the northern region of Portugal would become 63.63% of all analysed porpoises.

Future research is needed to assess changes in seasonal Iberian porpoise distribution and in possible imbalances in sex ratio and differences in age classes across different areas. Currently, no further inferences are possible considering the large number of stranded individuals to which no sex or age class was attributed.

Using the annual population abundance estimates obtained between 2011 and 2015 in Portugal [43], the PBR estimate (using ƒ = 0.5) indicates an annual “acceptable” removal between 10 porpoises in 2011 (CI = 4–26) and 29 porpoises in 2013 (CI = 14–60). Notice that if the PBRƒ for endangered or declining species had been used (ƒ = 0.1), the estimated PBR would be drastically lower. The number of stranded porpoises with evidence of fishery interaction was used to evaluate whether PBR was surpassed or not, even though 40% of the animals stranded in the considered period were excluded from the analysis. However, using the most conservative approach possible, on average, at least 207 porpoises were removed from the population due to fishery interaction each year between 2011 and 2015 (estimated from the number of porpoises identified as bycatch over the number of strandings evaluated for fishery interaction, rather than the total number of strandings—96 and 159 individuals, respectively). The minimum population removal estimates indicate that about 5.55% of the population was removed annually due to fishery interaction evaluated from strandings in Portugal (Table S2). If we only consider the number of stranded porpoises that were evaluated for fishery interaction evidence, the annual population removal estimate increases to 9.19% of the population (Table 2). Therefore, bycatch in Portuguese continental waters is well above the 1.7% removal threshold recommended by the Agreement on the Conservation of Small Cetaceans in the Baltic, North East Atlantic, Irish, and North Seas (ASCOBANS) [54]. Further bycatch monitoring programs are needed, including accurate estimates of fishing effort and control of IUU fishing boats operating in high suitability areas for porpoises [48]. 

Attempts to highlight the porpoise bycatch situation in the Iberian Peninsula have been made for years [48]. In 2020, the Scientific Committee of the International Whaling Commission (IWC) acknowledged the high bycatch porpoise mortality, and the possible need for emergency measures was noted.

Given the importance of fishing-related activities in the study region, the primary threat to the Iberian porpoise population has been identified as bycatch, and at the least, strict implementation of EU directives and fisheries legislation is crucial. Recently, the European Commission called on member states to “Adopt national measures or submit joint recommendations to the Commission to minimise by-catch (or reduce it to the level that enables the full recovery of the populations) by the end of 2023 in the case of the harbour porpoise in Iberian Atlantic” [55]. If we aim at halting the porpoise decline in Portugal and Spain, an integrated overarching action plan directed at the conservation of the Iberian porpoise is needed. This action plan should include several mitigation measures to decrease bycatch and to account for the cumulative effects of other threats posed by the currently emerging blue economy activities in the marine environment (including marine renewable energy infrastructures).

Meanwhile, there is an urgent need for changes in fishing, particularly in those fisheries with highest bycatch rates, namely the polyvalent fleet using gill and trammel nets and beach seines. Fishing effort management measures should be applied within legal frameworks that prevent fisheries from losing profitability, particularly because legal measures are needed within an effective timeframe.

## 5. Conclusions

Stranding data are essential to understand the impact of threats, particularly on small and isolated marine populations, and to support science-based conservation and management strategies. In the present study, an increasing number of porpoise strandings was detected in Galicia (Spain) and Portugal. In Portuguese waters, the estimated annual population removal due to bycatch during the evaluated period indicated that the potential biological removal estimates for harbour porpoises was exceeded. There are still many gaps in knowledge concerning the Iberian porpoise population (mostly concerning seasonal spatial distribution, age, reproduction parameters, and social structure and behaviour) which should be urgently addressed to understand the potential resilience of this already-depleted population. The present study highlights the urgent need for reliable transboundary conservation and monitoring efforts with appropriate legal support. 

## Figures and Tables

**Table 1:** Stranded porpoises registered by the Galician (GAL) and the Portuguese (PT) stranding networks, between 2000 and 2020. The number and percentage of individuals are presented by sex, age class and cause of stranding. The Portuguese data are further detailed between the northern and southern sectors (see Figure 1).

	GAL	PT	Total	PT North	PT South
Sex					
n_sex_	232	524	756	453	71
Male	113, 48.71%	209, 39.89%	322, 42.59%	191, 42.16%	18, 25.35%
Female	90, 38.79%	202, 38.55%	292, 38.62%	186, 41.06%	16, 22.54%
ni	29, 12.50%	113, 21.56%	142, 18.78%	76, 16.78%	37, 52.11%
Age Class					
n_age_	105	406	511	383	23
Foetus	2, 1.90%	17, 4.19%	19, 3.72%	17, 4.44%	0, 0.00%
Neonate	7, 6.67%	31, 7.64%	38, 7.44%	30, 7.83%	1, 4.35%
Calf	15, 14.29%	27, 6.65%	42, 8.22%	23, 6.01%	4, 17.39%
Juvenile	50, 47.62%	169, 41.63%	219, 42.85%	166, 43.34%	3, 13.04%
Adult	12, 11.43%	118, 29.06%	130, 25.44%	107, 27.94%	11, 47.83%
ni	19, 18.10%	44, 10.84%	63, 12.33%	40, 10.44%	4, 17.39%
n_excl_^1^	127	118	245	70	48
Cause of stranding					
n_cause_	83	281	364	264	17
Bycatch	34, 40.96%	137, 48.75%	171, 46.98%	136, 51.51%	1, 5.88%
Probable bycatch	6, 7.23%	34, 12.10%	40, 10.99%	32, 12.12%	2, 11.76%
Diseases	3, 3.61%	19, 6.76%	22, 6.04%	18, 6.82%	1, 5.88%
Other	8, 9.64%	30, 10.68%	38, 10.44%	28, 10.61%	2, 11.76%
ni	32, 38.55%	61, 21.71%	93, 25.55%	50, 18.94%	11, 64.71%
n_excl_^2^	149	243	392	189	54

ni, not identified; n_excl_, removed from the analysis due to: ^1^ inaccessible location or unavailability of the stranding team; ^2^ inaccessible locations, unavailability of the stranding team and advanced decomposition state.

**Table 2:** Minimum harbour porpoise annual mortality from bycatch (EAM_bycatch_) and annual population removal due to bycatch (APR, %) estimated from stranded individuals on the Portuguese coast between 2011 and 2015. Strandings, number of harbour porpoise strandings registered annually by the Portuguese national strandings network which were evaluated for bycatch evidence (decomposition state ≤ 3). N_bycatch_, number of porpoise strandings resulting from bycatch. EAM, estimated annual mortality using mortality rate (Mr = 0.18) [33] and population estimate (N) for the period 2011–2015 [43]. CIs, in brackets.

Period	Strandings	N_bycatch_	Estimated Annual Mortality (EAM)	Carcass Detection Rate (CDR)	Estimated Annual Mortality from Bycatch (EAM_bycatch_)	Annual Population Removal (APR)
			Mr × N	Strandings/EAM (%)	N_bycatch_/CDR	EAM_bycatch_/N (%)
2011	24	10	215 (82–564)	11.15 (4.25–29.24)	90 (34–235)	7.50 (2.86–19.66)
2012	15	6	539 (273–1065)	2.78 (1.41–5.50)	216 (109–426)	7.20 (3.64–14.22)
2013	18	10	577 (276–1209)	3.12 (1.49–6.53)	321 (153–672)	10.00 (4.77–20.95)
2014	26	12	298 (129–686)	8.74 (3.79–20.15)	137 (60–316)	8.31 (3.60–19.14)
2015	13	11	386 (166–899)	3.36 (1.45–7.82)	327 (141–761)	15.23 (6.55–35.45)
2011–2015	Mean: 19	Mean: 10	406 (232–711)	4.73 (2.70–8.29)	207 (118–363)	9.19 (5.25–16.10)

**Table 3:** PBR values for the harbour porpoise (CI, confidence intervals) in Portuguese waters between 2011 and 2015 using *f* = 0.5. Abundance (*N*) and the respective coefficient of variation (CV) [43] were used to obtain the minimum population abundance estimate (*N*_min_). A 4% maximum net recruitment rate (R_max_) was considered [44].

Period	*N* (CI)	CV	*N*_min_ (CI)	PBR (CI)
2011	1196 (456–3135)	0.5070	968 (376–2586)	10 (4–26)
2012	2995 (1516–5917)	0.3495	2718 (1376–5370)	27 (14–54)
2013	3207 (1531–6718)	0.3814	2860 (1366–5992)	29 (14–60)
2014	1653 (717–3809)	0.4327	1431 (621–3296)	14 (6–33)
2015	2147 (923–4997)	0.4386	1851 (796–4309)	19 (8–43)
2011–2015	2254 (1287–3949)	0.2199	2166 (1237–3795)	22 (12–43)

## Data Availability

The data presented in this study are available on request from the corresponding author. The data are not publicly available because they were obtained under particular data sharing protocols, and they are still in use by the corresponding author.

## Supplementary Materials

The following supporting information can be downloaded at: https://www.mdpi.com/article/10.3390/ani13162632/s1, Table S1: Parameters used for the determination of bycatch assessment of harbour porpoises in Portugal, based on strandings data; Table S2: Minimum harbour porpoise annual mortality from bycatch (EAM_bycatch_) and annual population removal due to bycatch (APR, %) estimated from stranded individuals in the Portuguese coast between 2011 and 2015. Total strandings, number of harbour porpoise strandings in Portugal, including porpoises not evaluated for bycatch; number of porpoises evaluated for bycatch evidence is shown in brackets). N_bycatch,_ number of porpoise strandings resulting from bycatch. EAM, Estimated Annual Mortality using Mortality rate (Mr = 0.18) [33] and Population estimate (*N*) for the period 2011–2015 [43]. Confidence intervals (CIs) are presented in brackets.
