# Supplementary material for: Assessment of Harbour Porpoise Bycatch along the Portuguese and Galician Coast: Insights from Strandings over Two Decades
Source: Animals (Basel). 2023 Aug 15;13(16):2632. doi: 10.3390/ani13162632 (PMC10451651; doi:10.3390/ani13162632)
Supplement: Supplementary file 1 [file animals-13-02632-s001.zip › animals-2521042-supplementary.pdf]

animals

MDPI

Supplementary material

Table S1. Parameters used for the determination of bycatch assessment of harbour porpoises in Portugal, based on stranding data

Variable	Definition	Formula
$N_{bycatch}$	Number of stranded animals with evidence of bycatch	
EAM	Estimated annual mortality	EAM = *Abundance x Mortality rate*
CDR	Carcass detection rates	CDR (%) = $\frac{Number\ of\ strandings}{EAM}$
$EAM_{bycatch}$	Estimated annual mortality due to bycatch	$EAM_{bycatch} = \frac{Nbycatch}{CDR}$
$APR_{strandings}$	Annual population removal based on strandings data	$APR_{strandings}$ (%) = $\frac{EAMbycatch}{Abundance}$

Table S2. Minimum harbour porpoise annual mortality from bycatch ($EAM_{bycatch}$) and annual population removal due to bycatch (APR, %) estimated from stranded individuals in the Portuguese coast between 2011 and 2015. Total strandings, number of harbour porpoise strandings in Portugal, including porpoises not evaluated for bycatch; number of porpoises evaluated for bycatch evidence is shown in brackets). $N_{bycatch}$, number of porpoise strandings resulting from bycatch. EAM, Estimated Annual Mortality using Mortality rate (Mr=0.18) [33] and Population estimate (*N*) for the period 2011-2015 [43]. Confidence intervals (CIs) are presented in brackets.

Period	Total strandings	$N_{bycatch}$	Estimated Annual Mortality (EAM)	Carcass detection rate (CDR)	Estimated Annual Mortality from bycatch ($EAM_{bycatch}$)	Annual Population Removal (APR)
			Mr x *N*	Total strandings/ EAM (%)	$N_{bycatch}$ / CDR	$EAM_{bycatch}$ / *N* (%)
2011	38 (24)	10	215 (82-564)	17.65 (6.73-46.30)	57 (28-193)	4.74 (1.25-8.56)
2012	22 (15)	6	539 (273-1065)	4.08 (2.07-8.06)	147 (74-290)	4.91 (3.30-12.89)
2013	26 (18)	10	577 (276-1209)	4.50 (2.15-9.43)	222 (106-465)	6.92 (4.70-20.63)
2014	50 (26)	12	298 (129-686)	16.80 (7.29-38.14)	71 (34-178)	4.32 (1.49-7.91)
2015	23 (13)	11	386 (166-899)	5.95 (2.56-13.84)	185 (94-508)	8.61 (4.17-22.56)
2011-2015	Mean: 32 (19)	Mean: 10	406 (232-711)	7.84 (4.47-13.73)	125 (71-219)	5.55 (3.17-9.72)
